# A novel anti-OX40 human monoclonal antibody that blocks OX40/OX40L signaling and depletes OX40^+^ T cells

**DOI:** 10.1007/s42995-025-00284-y

**Published:** 2025-04-07

**Authors:** Zhen Li, Lin Liu, Xiaobo Chen, Yanqing Wang, Yuxuan Wang, Yuxiu Zhang, Bingqiang Zhang, Xiao Wu, Muhammad Omer Iqbal, Jin Chen, Yuchao Gu

**Affiliations:** 1 School of Medicine and Pharmacy, Ocean University of China, Qingdao, 266003 China; 2 Jingyuan Biosciences (Suzhou) Co Ltd, Suzhou, 215000 China; 3 Qingdao Center of Technology Innovation for Shark Antibody Development, College of Biological Engineering, Qingdao University of Science and Technology, Qingdao, 266042 China; 4 Key Laboratory of Cancer and Immune Cells of Qingdao, Qingdao Restore Biotechnology Co Ltd, Qingdao, 266111 China; 5 Qingdao Central Hospital, University of Health and Rehabilitation Sciences, Qingdao, 266042 China; 6 Fatima Tu Zahara Department of Life Sciences, Muhammad Institute of Medical and Allied Sciences, Multan, 60000 Pakistan

**Keywords:** Atopic dermatitis, OX40, Phage display, Fully human antibody, Computer simulation, Epitope scanning

## Abstract

Tumor necrosis factor receptor superfamily member 4 (TNFRSF4), also known as OX40, plays a crucial role in the regulation of T-cell immune responses under normal physiological conditions. Abnormal expression of OX40 and its cognate ligand OX40L (TNFSF4) have been associated with various autoimmune diseases, indicating that blocking the OX40/OX40L pathway could be a promising strategy for the treatment of a broad range of T cell-mediated autoimmune diseases. Here, we screened and characterized a fully human anti-OX40 antibody (JY007) from a naïve human scFv phage library. JY007 has an affinity constant of 7.71 nmol/L and effectively inhibited the OX40-OX40L interaction at both molecular and cellular levels, with IC_50_ values of 1.088 and 10.12 nmol/L, respectively. Furthermore, JY007 demonstrated the ability to deplete activated T lymphocytes through antibody-dependent cellular cytotoxicity (ADCC) activity, with an EC_50_ of 5.592 pmol/L. The combination of ADCC and its antagonist activity against OX40 suggests potential efficacy in suppressing inflammatory responses mediated by the OX40/OX40L pathway. Additionally, we employed molecular docking, site-directed mutagenesis, and competitive ELISA to pinpoint the epitopes on OX40. The results revealed that JY007 binds to Pro^37^, Ser^38^, and Asp^40^ of OX40. Interestingly, we also found that the most potent anti-OX40 antibody drug in the clinical stage, KHK4083, binds to different OX40 amino-acid residues, including Asp^74^, Lys^82^, Asp^117^, Ser^118^, Tyr^119^, and Lys^120^. This divergence suggests that the novel monoclonal antibody JY007 holds promise as a potential therapeutic option for patients with atopic dermatitis and may find broad applications in the treatment of autoimmune diseases.

**Supplementary Information:**

The online version contains supplementary material available at 10.1007/s42995-025-00284-y.

## Introduction

Atopic dermatitis (AD), one of the most common skin diseases, is a chronic inflammatory skin condition characterized by recurrent erythema, pruritus, eczematous lesions, skin thickening, and inflammation (Badloe et al. 2020). Studies have reported a rising incidence and prevalence of clinically diagnosed atopic dermatitis in recent decades, significantly impacting the quality of life for 15%-30% of children and 2%-10% of adults worldwide (Laughter et al. 2021).

The development, progression, and chronicity of AD are understood to be influenced by the interplay of genetic and environmental factors. Factors such as the disruption of the epidermal barrier, pruritus, skin inflammation, and immune deregulation also contribute to the complex etiology of this condition (Bonamonte et al. 2019). AD is recognized as a bidirectional T-cell-mediated disease, with Th2 cells primarily involved in the acute and chronic stages, while Th1 cells contribute to the inflammatory response that affects the chronicity of the disease (Kader et al. 2021). The dysregulation of these immune responses can lead to the onset and persistence of AD symptoms.

OX40, also known as CD134 or TNFRSF4, belongs to the TNFR superfamily and is highly expressed in activated T cells (Calderhead et al. 1993). OX40 has a single identified ligand, OX40L (also known as CD252 or TNFSF4), which is expressed primarily on activated antigen-presenting cells (APCs), such as endothelial cells, dendritic cells, and activated B cells (Ohshima et al. 1997). Upon engagement of OX40 with OX40L, downstream intracellular pathways in T cells are triggered, leading to the transcriptional activation of nuclear factor kappa B (NF-κB) and nuclear factor of activated T cells (NF-AT) (Song et al. 2008; Vanamee and Faustman 2018). Consequently, OX40 signaling activates CD4^+^ and CD8^+^ T cells and controls immune responses during various stages of T cell activation (So and Ishii 2019). However, upregulation of OX40-OX40L can cause excessive activation and harm to the inhibitory function of Tregs, leading to immune dysfunction and facilitating the onset of autoimmune diseases (Webb et al. 2016), including bowel disease, asthma, and experimental autoimmune encephalomyelitis (EAE). Notably, the overexpression of OX40 in atopic dermatitis has garnered significant attention (Furue and Furue 2021; Le and Torres 2022).

In recent years, considerable progress has been made in the development of potential novel therapeutic monoclonal antibodies (mAbs) targeting the OX40 on activated T cells for the treatment of moderate to severe atopic dermatitis. For example, GBR 830 (Guttman-Yassky et al. 2019), a humanized IgG1 monoclonal antibody developed by Glenmark, blocks the interactions between OX40 and OX40L to inhibit T cell-mediated immune responses. Another promising candidate is rocatinlimab (formerly KHK4083) (Nakagawa et al. 2020), a fully humanized anti-OX40 monoclonal antibody that has entered clinical trials. Rocatinlimab has shown the ability to inhibit the OX40-OX40L pathway to suppress the activation and proliferation of effector T cells and reduce the number of activated OX40^+^ T cells by ADCC. Clinical trials have demonstrated the significant reduction of symptoms in patients with atopic dermatitis treated with these antibodies, highlighting the effectiveness of OX40-targeting antibodies for the treatment of autoimmune diseases like AD. Despite the promising results, some patients may not respond to treatment or discontinue it due to side effects (Guttman-Yassky et al.^2019^, ^2023^). Moreover, prolonged use of monoclonal antibody-based drugs may lead to the development of drug resistance in patients (Brand et al. 2011; Smith 2003).

Based on the challenges above, our study utilized a naïve human single-chain fragment variable (scFv) phage library to screen and characterize anti-OX40 antibodies. Among the identified antibodies, JY007, a fully human antibody targeting human OX40 and binding to distinct epitopes compared to rocatinlimab, exhibited comparable binding and functional activities. These findings suggest the potential of JY007 as a novel drug candidate for treating atopic dermatitis (AD) and other T-cell-mediated diseases.

## Materials and methods

### Screening of a human scFv phage display library against OX40

A naïve human scFv phage display library (1 × 10^10^ pfu/mL) was obtained from Jingyuan Biosciences (Suzhou) Co., Ltd. The panning experiment was similar to the previous descriptions (Xi et al. 2023). Recombinant OX40-His protein (Acro Biosystems, China, OX0-H5224) was incubated overnight at 4 °C in 96-well plates. After the wells were blocked by 3% BSA or 5% no-fat milk, the naïve human scFv phages were added and incubated for 1 h at 37 °C. The unbound phages were washed out with PBST (PBS with 0.1% Tween-20). Finally, OX40-specific binding phages were eluted with triethylamine (100 mmol/L) and used to infect *E. coli* TG1 cells for the production and purification of phages for the next screening round. The same screening procedure was repeated for three rounds.

### Polyclonal phage ELISA

After three rounds of panning, polyclonal phage ELISA was performed to identify the enrichment of phage antibodies against OX40-His. The OX40-His protein was diluted and incubated overnight at 4 °C in 96-well plates. After blocking with 5% no-fat milk at 37 °C for 1 h, the diluted phage from rounds 1, 2, and 3 (5 × 10^11^ pfu/mL) were added to the coated plates. Subsequently, 1:10,000 diluted HRP-conjugated anti-M13 (Sino Biological, China, 11,973-MM0) was added as the secondary antibody. Finally, TMB (Inno Reagents, TMB-S-003) was added to each well and incubated at 37 °C. The reaction was stopped by adding 1 mol/L H_2_SO_4,_ and the optical density (OD) of 450 was measured.

### Monoclonal phage ELISA

To obtain monoclonal phages against OX40-His, 96 colonies were selected from the third round of panning. Briefly, phages were added to 96-well plates coated with OX40-His and incubated at 37 °C for 1 h. The procedure for detecting bound phages was similar to that of the polyclonal phage ELISA. Wells with OD values three times higher than the control well were considered positive clones. The positive clones were selected for DNA sequencing analysis. The obtained sequences were then analyzed using IMGT V-quest (http://www.imgt.org/IMGTindex/V-QUEST.php) to identify the differences in the scFvs.

### Expression and purification of scFv antibodies

The above scFvs genes with His and HA tags from the pcomb3xss were amplified by PCR and cloned into pCDNA3.1. Recombinant plasmids were transformed into Trans5α cells (Yeasen Biotech, China, CD201-01), which were then cultured overnight in an LB medium with ampicillin. The plasmids were extracted using the Low Endotoxin Plasmid Small Extraction Medium Kit (Magen, P1154). The plasmids were then transfected into suspension-adapted ExpiCHO-S™ (Thermo Fisher Scientific, USA, A29127) cells by lipofectamine (Thermo Fisher Scientific, USA, A29133) to express the target proteins. On day 11, the supernatants were collected by centrifugation, and the scFvs were purified using a HisTrap HP column (GE Healthcare, USA, 29,051,021). The purified scFvs were then analyzed by sodium dodecyl sulfate–polyacrylamide gel electrophoresis (SDS-PAGE).

### ELISA binding assays

ELISA was performed for the evaluation of the scFvs binding to OX40. Briefly, 96-well plates were coated with OX40-His at 4 °C overnight. The next day, the plates were washed three times with 0.1% PBST and blocked with 3% BSA. Subsequently, serially diluted scFvs were added and incubated at 37 °C for 1 h. Then a 1:10,000 diluted HRP-conjugated anti-HA antibody (Sigma, 11,667,475,001) was used as the secondary antibody. The intensity of the absorbance signals was measured at 450 nm.

### Competitive ELISA assays

96-well plates were coated with OX40-His (100 ng/well in PBS) at 4 °C overnight. The diluted OX40L-Fc solutions were added to the plates and incubated at 37 °C for 1 h. Next, HRP-conjugated goat anti-human IgG (H + L) (Sino Biological, China, SSA002) was added and incubated at 37 °C for 1 h. OD values were read at 450 nm with Tecan Spark. For further analysis, 96-well plates were coated with OX40-His at 4 °C overnight and blocked with 3% BSA. Then serial dilutions of the scFvs were pre-mixed with 0.3 μg/mL OX40L-Fc before incubation and added to the coated plates. The same procedure as above was followed.

### Luciferase reporter assay for scFv antibodies

The HT1080 human OX40 cell line (Kangyuan Bochuang Biosciences, KC-0140) was applied to assess whether the scFvs can antagonize the OX40-OX40L interaction with the existence of OX40L. HT1080 human OX40 cells were harvested and added to a white 96-well plate, plated 1.2 × 10^6^ per well in 25 μL, and incubated with fivefold diluted A1, A8, A9, C8, G2, and G8 scFvs at 37 °C for half an hour. Then, HEK-293T human OX40L cells (Kangyuan Bochuang Biosciences, KC-0295) were harvested, and 2 × 10^4^ cells in 25 μL were added to the plate per well, and the plate was incubated at 37 °C for 4.5 h. Finally, the One-Lite Luciferase Assay System (Nanjing Novozymes, DD1203-01) was incubated for 5 min. Tecan Spark was used to detect the signal value.

### Expression and purification of IgG antibody

The JY007 was constructed by cloning the VH and VL into an expression vector that contained the human IgG1 constant region or Igκ constant region (Logen Biotech LLC). The plasmids containing the heavy- and light-chain Ig genes were co-transfected into ExpiCHO-S™ cells by lipofectamine. During the transfection process, 2-FF (a competitive substrate for fucosyltransferase) was added to remove core fucose from monoclonal antibody N-glycans to improve their antibody-dependent cellular cytotoxicity. The IgG1 expressed in CHO were purified using Hitrap protein A HP (GE Healthcare, USA, 29,048,576).

### Binding assay to human OX40

The binding of JY007 to OX40 was analyzed by ELISA. Briefly, 96-well plates were coated with OX40-His overnight at 4 °C. JY007 and KHK4083 were diluted and incubated at 37 °C for 30 min. For the detection of antibodies, HRP-conjugated goat anti-human IgG (H + L) secondary antibody was diluted at 1:10,000 in 3% BSA and incubated at 37 °C for 30 min. The plates were washed three times and incubated with TMB substrate at 25 °C, followed by adding a stop buffer. Finally, the intensity of the absorbance signals was measured at 450 nm.

### Species cross-binding activity

96-well plates were coated with the recombinant Cynomolgus monkey OX40 protein overnight at 4 °C. Cross-binding activities were detected using the same procedure as the binding assay for human OX40 by ELISA.

### Antibody affinity determination by biolayer interferometry

For analysis of mAbs binding to OX40-His on a GatorPrime from Gator Bio, JY007 or KHK4083 were captured at 100 nmol/L in KD buffer (PBS solution containing 0.02% Tween-20 and 0.1% BSA) on protein A probes. Kinetic measurements were performed by serial dilutions (50 nmol/L, 100 nmol/L, and 200 nmol/L) of OX40-His in KD buffer as an association step (90 s) and KD buffer as a dissociation step (180 s) according to the method described (Chen et al. 2023). The protein A probes were regenerated with glycine–HCL buffer, pH 1.9, for 15 s, repeated three times. Through the real-time data provided by GatorPrime, binding kinetics (on-rate and off-rate) and affinity values (dissociation constant) were calculated by the GatorOne software v2.10.

### Blockade of OX40L to OX40

A competitive ELISA assay was used to determine whether JY007 could block the interaction between recombinant OX40 to OX40L-mFc. 96-well plates were coated with OX40-His at 1 μg/mL overnight at 4 °C. After the wells were blocked with 3% BSA, serial dilutions of JY007 were pre-mixed with 0.3 μg/mL OX40L-mFc and added to the wells. Following 1 h of incubation, the plates were washed and incubated with 1:10,000 HRP-conjugated goat anti-mouse IgG (H + L) (Sino Biological, China, SSA021) at 37 °C. The plates were washed and incubated with TMB substrate, followed by adding a stop buffer (1 mol/L H_2_SO_4_). OD values were read at 450 nm with Tecan Spark.

### Western blotting assays

To analysis whether the downstream NF-κB signaling pathways were altered by JY007 antagonism, human OX40-expressing HT1080 cells were harvested and incubated with 100 nmol/L JY007 or KHK4083 at 37 °C for 10 min. Then the 100 ng/mL OX40L-mFc was added and incubated at 37 °C for 30 min. Following the method of Liu (Liu et al. 2024a, b), the cells were lysed in RIPA Lysis Buffer. SDS-PAGE electrophoresis was performed and the protein transferred from the gel to a PVDF membrane. Phospho-NF-κB p65 Rabbit mAb (CST, CST3033) or NF-κB p65 XP® Rabbit mAb (CST, CST8242) was used for immunoblotting, then incubated with HRP-conjugated goat anti-rabbit IgG antibody (Abcam, ab6721). All blots were developed with ultra-sensitive horseradish in the catalase DAB color kit (Sangon Biotech, Shanghai, C510023).

### Luciferase reporter assay of IgG antibody

To perform the luciferase assay, the human OX40-expressing HT1080 cell line was applied to assess whether JY007 could antagonize the OX40-OX40L interaction. The method was similar to the luciferase reporter assay for scFvs, with the difference that the antibodies of JY007, KHK4083, and IgG1 were fivefold diluted and started at a concentration of 30 μg/mL. Tecan Spark was used to detect the signal value.

### Flow cytometry assay

Total T cells (Milestone® Biotechnologies, TPCS#PB03-N-1C) were thawed in 1640 medium supplemented with 10% FBS and centrifuged at 400 g for 10 min. The total T cells were then resuspended in the prepared buffer (RPMI 1640 + 10% FBS + 1% AA) and diluted to 1 × 10^6^ /mL. The total T cells were activated at 37 °C for 3 days with 5 μg/mL of PHA (Roche, 11,249,738,001) and 20 ng/mL of hIL-2 (Farcet et al. 1984) (Sino Biological, China, GMP-11848-HNAE). Furthermore, JY007, KHK4083, and OX40L-mFc were incubated with the activated T cells at 4 °C for 1 h. 1:1000 FITC goat anti-mouse IgG H&L (Abcam, China, Ab7064) or 1:500 FITC anti-human IgG (Abcam, China, Ab7149) was used to label the OX40L-mFc, JY007, or KHK4083 binding to the OX40 expressed on the activated T cells.

To block OX40 costimulation of T-cell proliferation, the JY007 mAb was functionally screened for its ability to inhibit the OX40-OX40L interaction. The activated T-cells were plated at 10,0000 cells/50 μL per well in a 96-well V bottom plate. JY007 or KHK4083 was diluted to a starting concentration of 200 nmol/L and mixed with 0.2 μg/mL OX40L-mFc and incubated with the activated T cells at 4 °C for 1 h. The OX40L-mFc was fluorescently labeled with FITC goat anti-mouse IgG H&L. Data were analyzed using FlowJo.

### ADCC assay

Target cells (Target, T: HT1080 human OX40 cells) were harvested and labeled with Cell Trace Far-Red dye (Thermo Fisher Scientific, USA, C34572) for 2 days, then seeded at a concentration of 2 × 10^4^ cells per well in a 96-well U bottom plate and incubated with 0.0000512–100 nmol/L of JY007, KHK4083, or control IgG1 for 30 min on ice. 1.2 × 10^6^ PBMCs per well (Effector: E; Miosheng Biotech) at E/T = 60/1 were added to the wells in a final volume of 100 μL. After incubation for 4.5 h at 37 °C, 5 μL of PI dye (Sangon Biotech, Shanghai, E60730) was added to the mixed cell solution to label dead cells and incubated for 2–3 min at 25 °C. The samples were analyzed by Flow Cytometry (CytoFLEX).

### Epitope-blocking ELISA assay

A competitive ELISA was performed to analyze the binding epitopes of the JY007 and KHK4083 on OX40. Briefly, 96-well plates were coated with JY007 (100 ng/well) overnight at 4 °C. Serial dilutions of KHK4083 were pre-mixed with 0.2 μg/mL OX40L-His before incubation in coated wells. The HRP-conjugated anti-His (Sigma, China, 105,327-MM02T-H) was used as the secondary antibody and diluted 1:10,000 in 3% BSA, and the plates were then incubated with TMB substrate at 25 °C for 10 min. OD values were read at 450 nm with Tecan Spark.

### Alanine scanning

The crystal structure of the human OX40-OX40L complex has been characterized in detail by Compaan and Hymowitz (Compaan and Hymowitz 2006). The key residues of the KHK4083/OX40 and JY007/OX40 interface were studied by performing alanine scanning mutagenesis. A total of 17 OX40 variants cloned into pCDNA3.1 were synthesized (Logen Biotech LLC). Next, we accessed the affinities of the JY007 and KHK4083 binding to the individual OX40 mutants and the wild-type OX40 by BLI. Briefly, OX40 mutants were loaded onto anti-His probes and OX40-His protein at the indicated concentration (100 nmol/L) as a positive control. Next, 100 nmol/L KHK4083 or JY007 was used for association and then dissociated in KD buffer. The anti-His probes were regenerated with glycine–HCL buffer, pH 1.9, repeated three times. Through the real-time data provided by GatorPrime, binding kinetics (on-rate and off-rate) and affinity values (dissociation constant) were calculated by the GatorOne software v2.10.

### Computational model

The amino acid sequence of OX40 (P43489) was obtained from the Uniprot database. The structures of OX40-KHK4083 and OX40-JY007 3D complexes were constructed using ColabFold (Mirdita et al. 2022). ColabFold is a deep learning model based on AlphaFold (Mirdita et al. 2022), which has better user friendliness, lower computational resource requirements, and high-precision protein structure prediction. Each composite structure was predicted to generate 25 binding models, among which the model with the highest binding energy and the most reasonable structure was selected and used for subsequent analysis. The molecular docking was as described previously (Liu et al. 2024a, b).

### Confirmation of the 3D structure of OX40-JY007

OX40 mutations with alanine substitutions, OX40-18 and OX40-19, were constructed by Logen Biotech LLC and expressed using CHO cells. The affinities of antibodies JY007 and KHK4083 binding to the individual OX40 mutants were detected by the same procedure as above.

## Results

### Screening of Anti-OX40 antibodies from naïve human scFv phage library

**Fig. 1:**
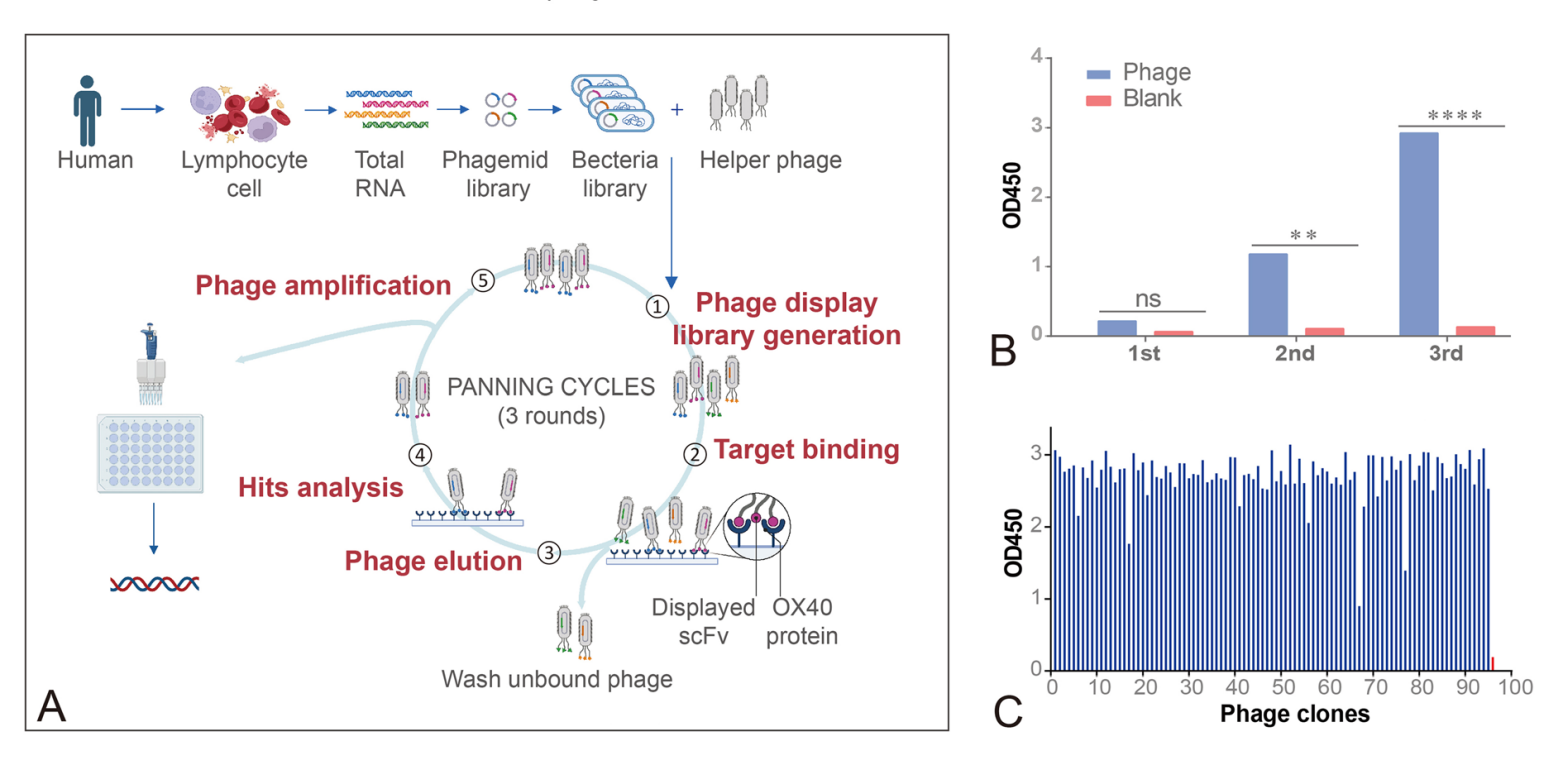
Selection of anti-OX40 scFv antibodies. **A** Schematic illustration of the construction and screen of a naïve scFv phage display library. Figure created by BioRender. **B** Polyclonal phage ELISA. The phage that specifically binds to OX40 was enriched in the third round. Error bars represent the standard deviation. Data are presented as mean ± SD. *P < 0.05, **P < 0.01, ***P < 0.001, ****P < 0.0001, versus control. **C** Monoclonal phage ELISA. Most monoclonal phages (blue bar) that bound with high affinity to OX40 and PBS buffer (red bar) were used as negative controls. The positive signal is defined as being three times higher than the negative signal

Based on a naïve human scFv phage library, the OX40-binding phages were selected by three rounds of solid-phase screening (Fig. 1A). Polyclonal phage ELISA was used to detect the enrichment of the phages binding to OX40-His. The signals gradually increased from the initial library to the third round, and the control signal was always kept low, indicating the enrichment of the binding-specific scFv phages (Fig. 1B). We then randomly picked up 96 single colonies for the monoclonal phage ELISA (Fig. 1C), and clones with a high binding capacity to OX40 were identified by sequencing. Finally, six scFv antibodies with different sequences were found to be effectively enriched and named A1, A8, A9, C8, G2, and G8 (Supplementary Fig. 1A and B).

### Functional characterization of the anti-OX40 scFv antibody in vitro

**Fig. 2:**
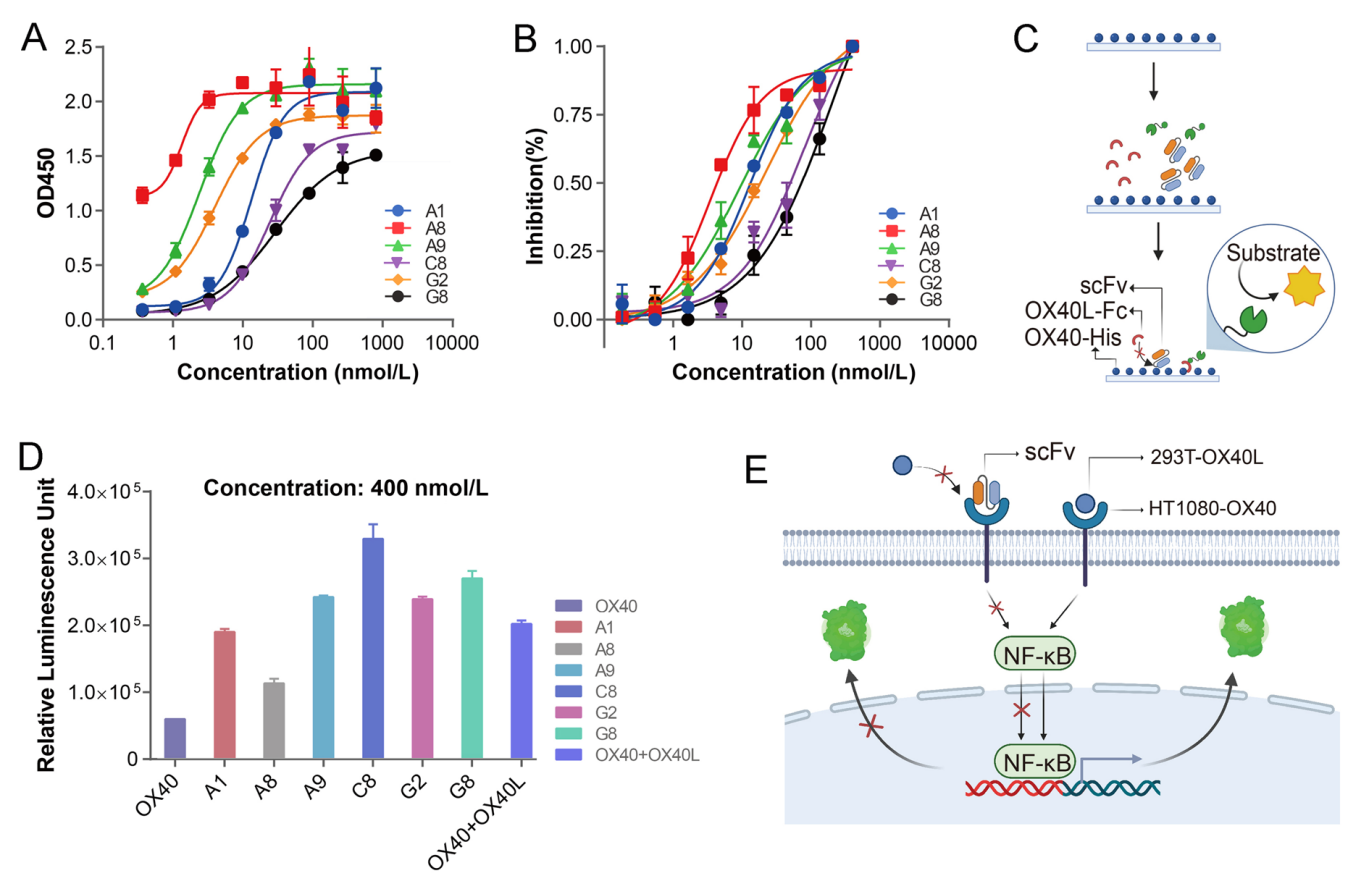
The binding activity of anti-OX40 scFv antibodies to human OX40. **A** Binding of scFv antibodies to OX40 measured by ELISA. Gradient dilutions of scFv antibodies and 1 μg/mL OX40 and anti-HA as secondary antibody. **B** Competitive ELISA between OX40 and scFv antibodies and OX40L. Gradient dilutions of scFv antibodies were incubated with precoated OX40 and OX40L-Fc. Error bars represent standard deviation. Data are presented as mean ± SD, *n* = 6 in triplicate. **C** Luciferase reporter assay between OX40 and scFv antibodies and OX40L.The HT1080-OX40 reporter cells were co-cultured with HEK-293T-OX40L cells and anti-OX40 scFv antibodies, followed by the determination of luciferase activity. Error bars represent standard deviation. All data are presented as mean ± SD, *n* = 6 in triplicate. All schematic illustrations were created by BioRender

The six scFv antibodies were expressed in CHO-S cells and purified by Ni–NTA column (Supplementary Fig. S2A). To determine its binding affinities, the EC_50_ values of the anti-OX40 antibodies were evaluated using indirect ELISA (Fig. 2A), showing A8 exhibiting the strongest binding activity with an EC_50_ of 1.33 nmol/L. To assess the inhibitory potency of anti-OX40 scFv antibodies, we initially determined the EC_50_ values of OX40-His binding to OX40L-Fc. Subsequently, different concentrations of scFv antibodies were used to antagonize the OX40-OX40L interaction (Fig. 2B). The results indicated that among the six scFv antibodies, A8 exhibited the highest inhibitory activity, with an IC_50_ value of 3.46 nmol/L. Luciferase assays are an invaluable tool for the identification and characterization of functional variants, enabling the study of intracellular signaling pathways (Nair and Baier 2018). To identify the effectiveness of these antibodies, we established a cell-based luciferase reporter assay to assess the blocking effect of anti-OX40 scFv antibodies. As shown in Fig. 2C, A8 demonstrated potent blocking of the OX40-dependent NF-κB signal pathway. Together, these findings demonstrate that the scFv antibody A8 substantially inhibits the interaction between OX40 and OX40L at both the molecular and cellular levels, thus warranting further investigation.

### Antibody binding activity of JY007

**Fig. 3:**
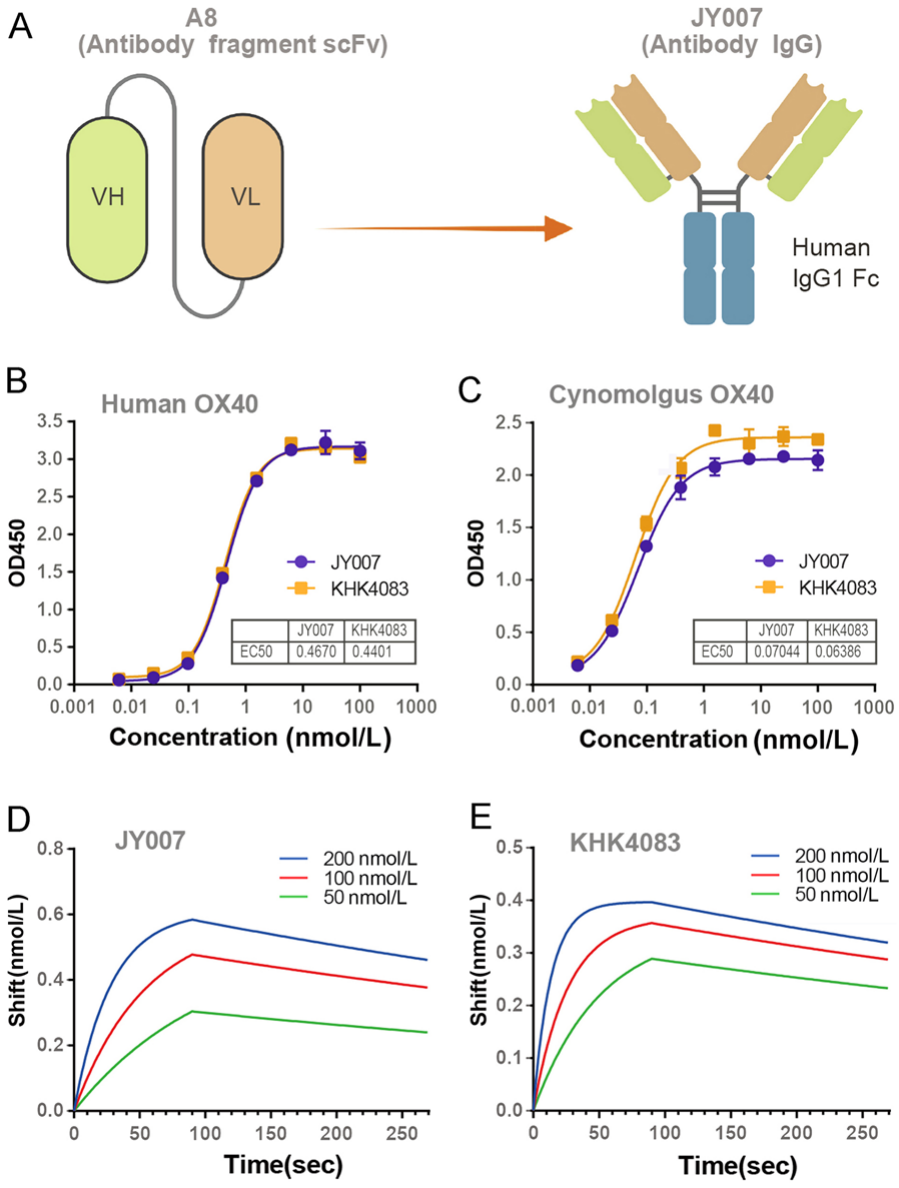
The binding activity of JY007 and KHK4083. **A** The structure of antibodies A8 and JY007. Schematic illustration created by BioRender. **B** ELISA assay for the binding activity of JY007 to human OX40. Gradient dilutions JY007 and 0.5 μg/mL human OX40 and goat anti-human IgG (H + L) secondary antibody. Error bars represent standard deviation. Data are presented as mean ± SD, *n* = 6 in triplicate. **C** ELISA assay for the binding activity of JY007 to Cynomolgus OX40. Gradient dilutions of KHK4083 and 1 μg/mL Cynomolgus OX40 and goat anti-mouse IgG (H + L) secondary antibody. Error bars represent standard deviation. Data are presented as mean ± SD, *n* = 6 in triplicate. **D** The binding kinetic plots of antibody JY007 were measured by BLI. JY007 was set at 100 nmol/L and OX40 was applied at concentrations ranging from 50 to 200 nmol/L. **E** The binding kinetic plots of antibody KHK4083 were measured by BLI. KHK4083 was set at 100 nmol/L and OX40 was applied at concentrations ranging from 50 to 200 nmol/L

Based on the results above, we constructed an IgG format of A8 (named JY007) and expressed it in CHO-S cells (Fig. 3A). The SDS-PAGE assay was used to analyze the purified antibodies, and the results showed that the apparent molecular weight was 150 kDa in size. Upon reduction, the apparent molecular weights of the heavy and light chains were 50 and 25 kDa, respectively, with a purity of over 90% (Supplementary Fig. S2B). Then, we further evaluated the binding activities of JY007 to human OX40 and Cynomolgus OX40. The ELISA results showed that JY007 demonstrated a high binding affinity to human OX40 and Cynomolgus OX40, similar to KHK4083 (Fig. 3B and C). Finally, BLI was used to measure the affinity of JY007 binding to human OX40 with a K_D_ of 7.71 nM compared to a K_D_ of 3.24 nM from KHK4083 (Fig. 3D and E, Table 1).

**Table 1:** Analysis of the affinity kinetics of anti-OX40 antibodies by BLI

Antibodies	K_on_ (1/ms)	K_off_ (1/s)	K_D_ (M)
JY007	1.71 × 10^5^	1.32 × 10^–3^	7.71 × 10^–9^
KHK4083	3.72 × 10^5^	1.2 × 10^–3^	3.24 × 10^–9^

### Antibody JY007 inhibits the OX40 interaction with OX40L

**Fig. 4:**
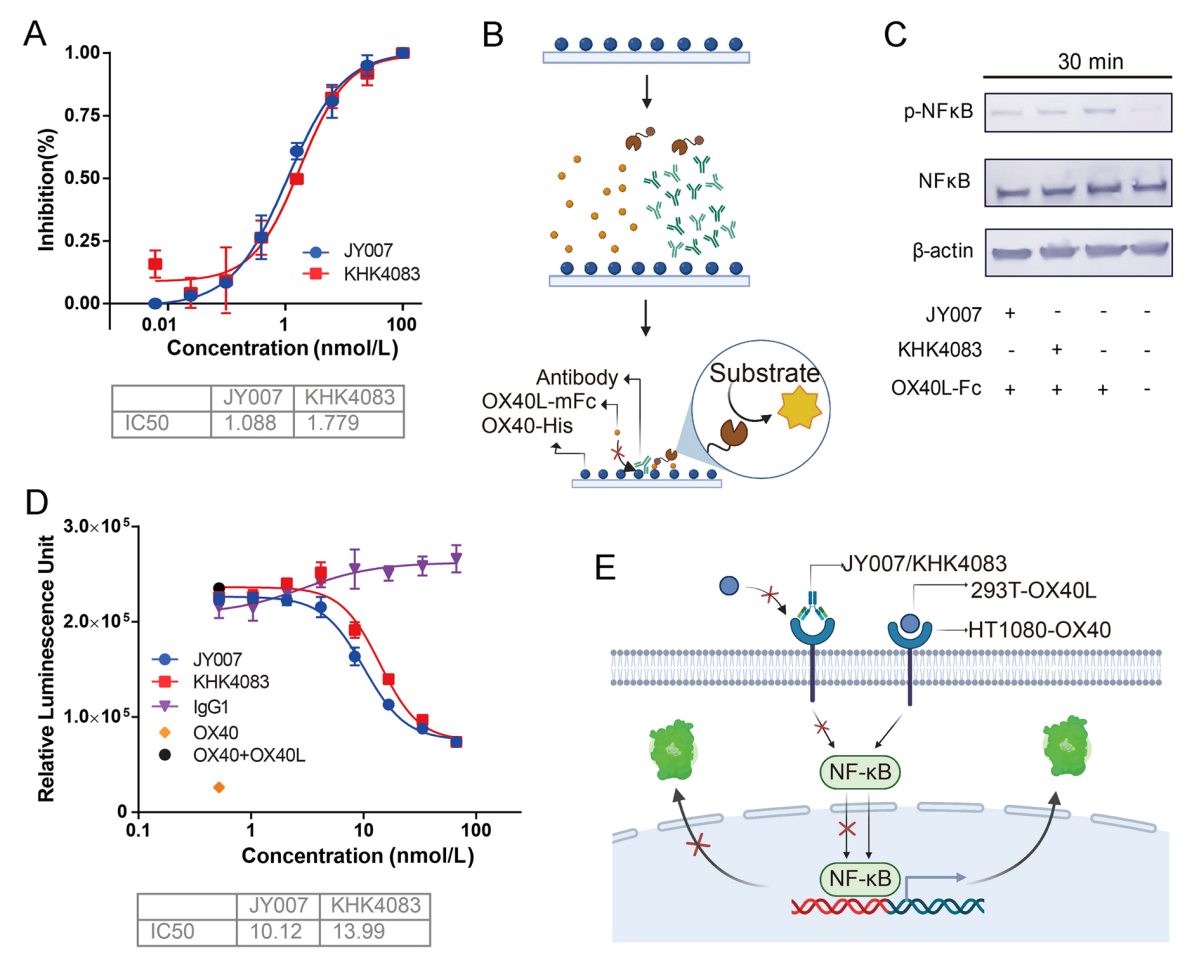
Antibody JY007 blocks the OX40 interaction of OX40L. **A** Competitive ELISA between OX40, JY007, and OX40L. Gradient dilutions of JY007 were incubated with precoated OX40 and OX40L-mFc, with HRP-conjugated goat anti-mouse IgG (H + L) as the second antibody. KHK4083, Kirin Pharmaceutical Co., was used as a positive control. Error bars represent standard deviation. Data are presented as mean ± SD, *n* = 6 in triplicate. **B** Western blotting assays. HT1080-OX40 reporter cells were co-cultured with 100 nmol/L JY007 or KHK4083 for 10 min and treated with 100 ng/mL OX40L-mFc for 30 min, western blotting with Phospho-NF-κB p65 Rabbit mAb as first antibody, with HRP-conjugated goat anti-rabbit IgG as second antibody. Beta-actin served as a loading control. **C** Luciferase reporter assay between OX40 and JY007 or OX40L. HT1080-OX40 reporter cells were co-cultured with HEK-293T-OX40L cells and JY007, followed by the determination of luciferase activity. KHK4083 was used as a positive control. Error bars represent standard deviation. Data are presented as mean ± SD, *n* = 6 in triplicate. All schematic illustrations were created by BioRender

Competitive ELISA was used to examine the ability of JY007 to block the interaction between OX40 and OX40L. JY007 antagonized the interaction of OX40L with human OX40 with an IC_50_ of 1.088 nmol/L (Fig. 4A), which was about 1.63 times stronger than that of KHK4083. Engagement of OX40 and OX40L for co-stimulatory molecules by the APCs is a key regulator of T cell responses and viability through the NF-κB pathway that controls the activity of intracellular targets for promoting the co-stimulatory signal regulating proliferation and survival of T cells (Song et al. 2008; Arch and Thompson 1998). NF-κB activation was then determined by western blotting. JY007 and KHK4083 were co-cultured with OX40 cells for 10 min, and then treatment with OX40L for 30 min was able to influence the activation of NF-κB (Fig. 4B), indicating that JY007 inhibits the OX40-OX40L interaction, thus affecting the NF-κB activation. HT1080-OX40 cells with NF-κB luciferase reporter were co-cultured with different concentrations of anti-OX40 antibody and OX40L cells. Compared with OX40L, antibody treatment inhibited the reporter activity with an IC_50_ of 10.12 nmol/L, indicating that it can block the activation of NF-κB signaling in the assay system (Fig. 4C). And the antibody JY007 was approximately 1.38 times stronger than that of KHK4083, which is consistent with the result of the western blot assay.

Antibody JY007 induces OX40^+^ T cell depletion.

**Fig. 5:**
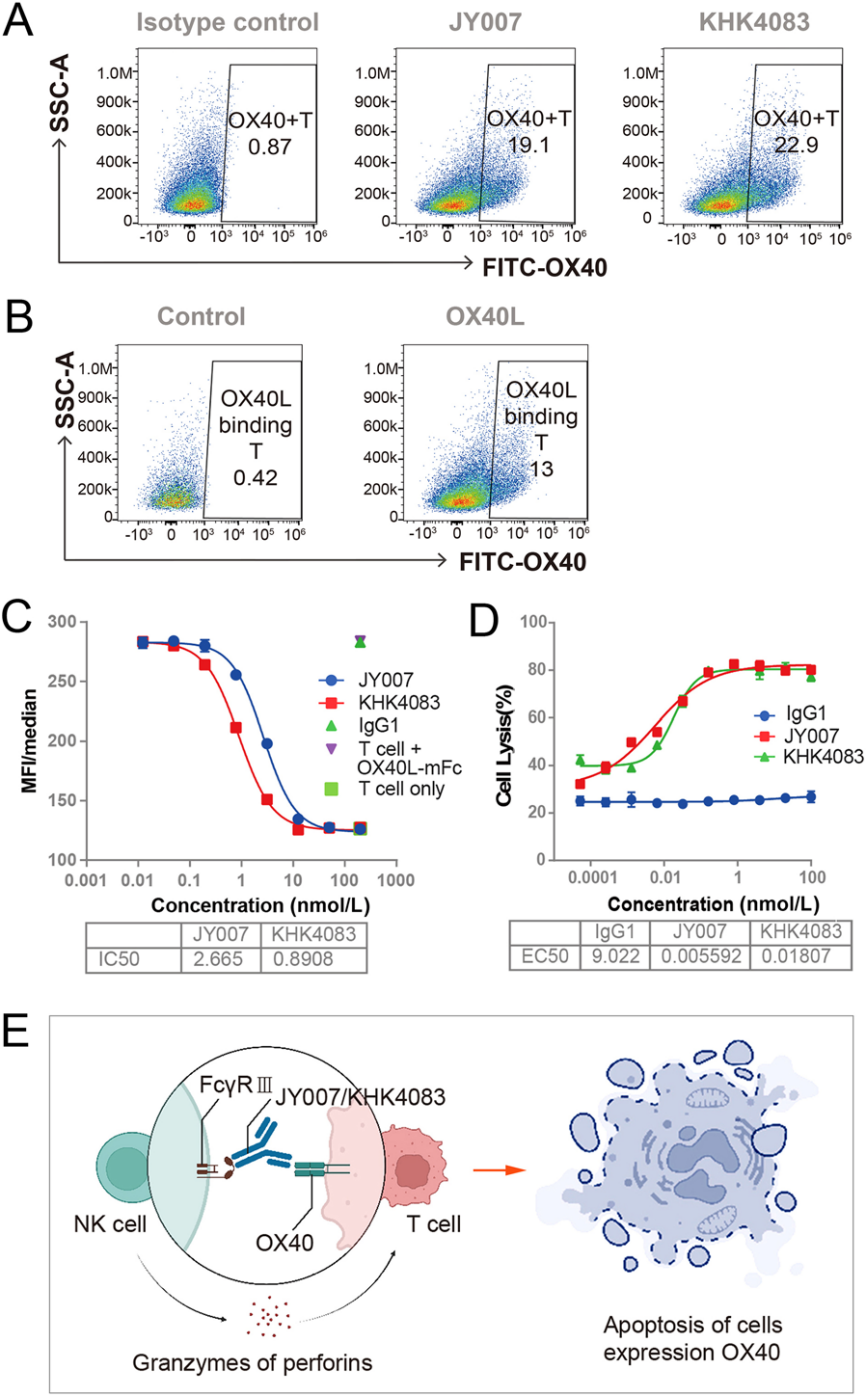
Antibody JY007 induces OX40 positive T cell depletion. **A** The binding of active T cells to antibodies. T cells were activated at 37 °C for 3 days with 5 μg/mL PHA and 20 ng/mL hIL-2. JY007 and KHK4083 were incubated with the activated T cells at 4 °C for 1 h, with FITC anti-human IgG as a label antibody. **B** The binding of active T cell to OX40L. OX40L-mFc were incubated with the activated T cells at 4 °C for 1 h, with FITC anti-human IgG as label antibody. **C** The flow cytometry blocking assay of JY007. Gradient dilutions of JY007 were incubated with activated T cells and 0.2 μg/mL OX40L-mFc, with FITC anti-mouse IgG (H + L) was used as the secondary antibody. KHK4083 as a positive control. Error bars represent standard deviation. Data are presented as mean ± SD, *n* = 6 in triplicate. **D** ADCC assay between JY007 and OX40 cells and PBMC. Gradient dilutions of JY007 were incubated with 2 × 10^4^ target cells (HT1080-OX40-NF-κB-luc2) and 1.2 × 10^6^ effector cells (PBMC); E/T = 60/1. KHK4083 as a positive control. Error bars represent standard deviation. Data are presented as mean ± SD, *n* = 6 in triplicate. Schematic illustration created by BioRender

To investigate the function of JY007 at the cellular level, we examined the expression of OX40 on the activated T cells. As shown in Fig. 5A and B, activated T cells can bind to KHK4083, JY007, and OX40L. Flow cytometry was used to further the study, and JY007 effectively inhibited the OX40-OX40L interaction with IC_50_ values of 2.665 nmol/L (Fig. 5C). Furthermore, JY007 and KHK4083 induce the deletion of OX40^+^ T cells via FcγR-mediated induction of antibody-dependent cellular cytotoxicity (ADCC), with EC_50_ values of 5.592 and 18.07 pmol/L, respectively (Fig. 5D). These results revealed that the functional activity of JY007 was stronger than that of KHK4083, which can potentially block OX40 signaling and induce depletion of cells such as effector T cells that express high levels of OX40.

### JY007 and KHK4083 have different binding epitopes on OX40

**Fig. 6:**
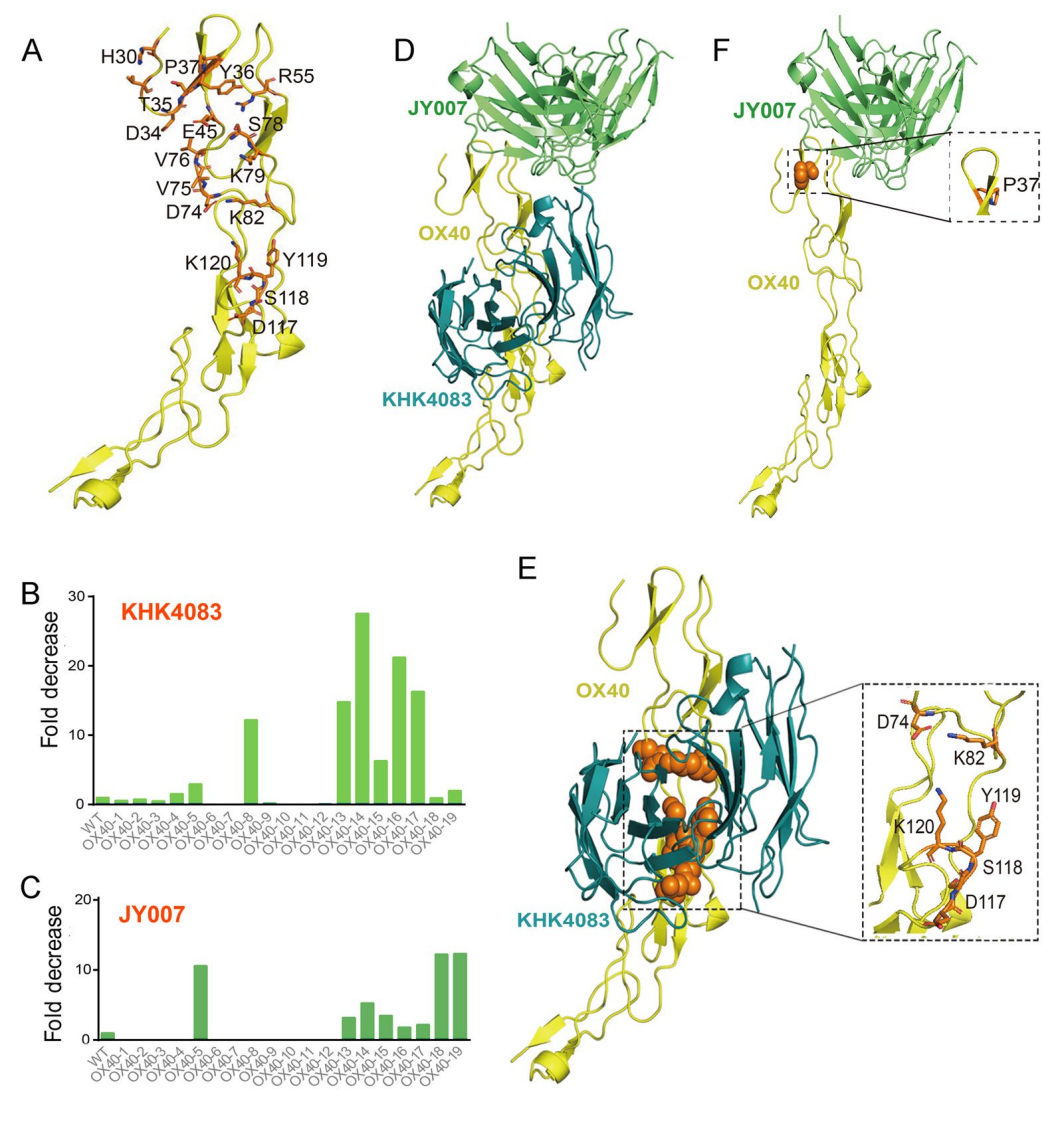
Epitope scanning for hotspot identification and model docking. **A** Epitope scanning. Seventeen key OX40 residues were mutated to alanine, and their binding to the antibody was tested. **B** Fold decrease in the affinity of KHK4083 for different OX40 mutants. The binding activity of 100 nmol/L KHK4083 to the OX40 mutants. The fold decrease was calculated from the BLI of the KHK4083-OX40 interaction by comparing the K_D_ value of each mutant with wild-type OX40. Five mutations (D74, K82, D117, S118, Y119, and K120) significantly affect the fold decrease. **C** Fold decrease in the affinity of JY007 for different OX40 mutants. The binding activity of 100 nmol/L JY007 for the OX40 mutants. The fold decrease was calculated from the BLI of the JY007-OX40 interaction by comparing the K_D_ value of each mutant with wild-type OX40. P37 significantly affects the fold decrease. **D** The 3D structure of OX40 binding to KHK4083 and JY007. KHK4083 (blue) and JY007 (green) bind to the different epitopes of OX40 (yellow). **E** The 3D structure of OX40-KHK4083. The five key positions of the affinity-changing residue (D74, K82, D117, S118, Y119, and K120) have been identified and mapped to the KHK4083 binding interface. **F** The 3D structure of OX40 binding to JY007. P37 was identified as a key affinity-modifying residue position and mapped to the JY007 binding interface

To determine the epitopes on OX40 recognized by JY007, we first evaluated whether JY007 and KHK4083 bound to the same epitopes on OX40 by competitive ELISA. The results showed that JY007 had no competition with KHK4083 binding to OX40 (Supplementary Fig. S3). Alanine scanning mutagenesis aims to identify those residues that contribute the most to the free energy of interaction by sequentially mutating residues in a protein of interest to alanine (Cunningham et al. 1989). Therefore, we first used alanine scanning to mutate key functional amino acids in OX40 to refine the epitopes of JY007 and KHK4083 (Fig. 6A; Supplementary Table 1) and evaluated the binding of JY007 and KHK4083 to OX40 mutations. As a result, the binding of KHK4083 to the mutants OX40-8, OX40-13, OX40-14, OX40-15, OX40-16, and OX40-17 was dramatically reduced compared to its binding to wild-type OX40 (Fig. 6B; Supplementary Table 2). In particular, the binding of JY007 to these mutants was less affected, except OX40-5 (Fig. 6C; Supplementary Table 3). These data indicated that the epitope binding regions of JY007 and KHK4083 on OX40 are different. Next, we used molecular modeling to explore the binding mode of JY007 or KHK4083, targeting OX40. In the OX40-JY007 and OX40-KHK4083 binding models (Fig. 6E and F), the two antibodies, JY007 and KHK4083, both occupy the OX40L binding site, but the two themselves are non-competitive, consistent with the competition experiments mentioned above (Fig. 6D). To further validate the 3D model of OX40-JY007, we evaluated the binding of JY007 to OX40 mutations, including Ser^38^ and ASP^40^. The binding of JY007 to these mutations was dramatically reduced compared to its binding to wild-type OX40. In contrast, the binding of KHK4083 to these mutants was less affected (Fig. 6B; Supplementary Table 3; Fig. 6C; Supplementary Table 3). By combining the computer simulations and experiments above, we have established a binding model for JY007 against OX40, which provides a theoretical basis for the subsequent development of novel antibodies against OX40.

## Discussion

The OX40-OX40L axis may enhance cytokine production (such as IL-2 and IL-4) and facilitate antigen-specific memory T cell proliferation, survival, and expansion (Yan et al. 2013). Therefore, an agonistic mAb targeting co-stimulating receptor OX40 on T cells, one of the most promising targets, could promote an antitumor immune response by inducing tumor anti-specific CD8^+^ T-cell responses and decreasing immunosuppressive regulatory T cells in vivo (Ruby et al. 2007). Stimulation of OX40 has been shown to increase antigen-specific effector T cell responses and patient-specific memory T cell production for therapeutic immunization strategies in cancer to prolong patient survival (Weinberg et al. 2000; Sadun et al. 2008).

By contrast, an antagonistic antibody against OX40 on T cells may suppress autoantigen-specific T cell responses and reduce immune activities in autoimmunity diseases by blocking OX40 signaling. Some studies have shown that in vivo blockade of OX40 signaling specifically suppresses the function of recently activated autoantigen-specific T cells, resulting in inhibition of autoimmune disease without severe immunosuppression (Li et al. 2007; Malmstrom et al. 2001; Nohara et al. 2001). Current OX40-based antibody therapy for moderate or severe atopic dermatitis has demonstrated remarkable success (Le and Torres 2022; Newsom et al. 2020). Nevertheless, there have been instances where patients have withdrawn from clinical trials due to adverse reactions associated with the use of these antibody drugs (Guttman-Yassky et al. 2019, 2023). Furthermore, prolonged exposure to monoclonal antibody drugs can lead to the development of drug resistance, thus limiting their effectiveness (Moccia et al. 2020; Martinez-Navio et al. 2020). Therefore, there is an urgent need for a new anti-OX40 antagonist antibody to meet the increasing medical demands of patients.

In this study, we screened a naïve human scFv phage library against OX40 and discovered that scFv antibody A8 exhibits a high affinity for OX40 and effectively blocks the OX40-OX40L interaction. Research demonstrated that a bivalent immunoglobulin G (IgG) format can improve scFv antibody particular effector function, half-life, and avidity (Sterner and Zehetmeier 2017), so we constructed an IgG1 antibody based on the genes of A8 VH and A8 VL and named it JY007 for further research. Our findings showed that JY007 binds to OX40 with high affinity, approximately 3 times higher than the scFv format, likely due to its conventional bivalent format.

In patients with atopic dermatitis, OX40 is highly expressed in damaged skin and is associated with antigen-specific T cells (Cavanagh and Hussell 2008), and the OX40L-OX40 axis plays a role in the sustained activation and expansion of effector T cells and effector memory T cells, which can have lasting effects on immune function (Furue and Furue 2021). Our results demonstrated that JY007 has comparable activities to KHK4083, with strong blocking activity against OX40 signaling and the ability to induce OX40^+^ T cells depletion. This suggests the potential efficacy of JY007 in treating patients with atopic dermatitis caused by unregulated OX40 signaling.

Molecular docking has become a fast alternative for studying the molecular basis of antigen–antibody interactions (Yang et al. 2019). Docking methods can be classified into two categories according to the sampling strategy applied during the simulation. The first class includes exhaustive searches for different antigen–antibody conformations without using knowledge of interface residues, the so-called ab initio docking algorithm and methods. The second includes data-driven docking algorithms that use predicted or experimentally determined epistasis and parity constraints to guide the search process (Ambrosetti et al. 2020). In our study, we performed data-driven docking to predict the structural interfaces between OX40 and JY007 or KHK4083. These predictions were based on functional assays of JY007, competitive ELISA between JY007 and KHK4083, and alanine scanning data. Interestingly, we found that JY007 binds to Pro^37^, Ser^38^, and Asp^40^ residues of OX40; whereas, KHK4083 binds to Asp^74^, Lys^82^, Asp^117^, Ser^118^, Tyr^119^, and Lys^120^ residues. These results indicated that JY007 and KHK4083 target different epitopes on OX40. The function of JY007 provided a solution for the drug resistance caused by KHK4083 in AD therapy, providing more choices for AD patients. In further studies, JY007 will be further evaluated through animal experiments to assess its therapeutic effect on atopic dermatitis.

## Supplementary Information

Below is the link to the electronic supplementary material.

Supplementary file1 (DOCX 1626 KB)

## Data Availability

The GenBank submissions staff and have received confirmation of the email address. The nucleotide sequences already available on GenBank: BankIt2731580 A1_VL; OR405855; BankIt2731729 A1_VH; OR405861. BankIt2731580 A8_VL; OR405856; BankIt2731729 A8_VH; OR405862. BankIt2731580 A9_VL; OR405857; BankIt2731729 A9_VH; OR405863. BankIt2731580 C8_VL; OR405858; BankIt2731729 C8_VH; OR405864. BankIt2731580 G2_VL; OR405859; BankIt2731729 G2_VH; OR405865. BankIt2731580 G8_VL; OR405860; BankIt2731729 G8_VH; OR405866.
