# Supplementary material for: A novel anti-OX40 human monoclonal antibody that blocks OX40/OX40L signaling and depletes OX40+ T cells
Source: Mar Life Sci Technol. 2025 Apr 7;7(2):328–39. doi: 10.1007/s42995-025-00284-y (PMC12102438; doi:10.1007/s42995-025-00284-y)
Supplement: Supplementary file 1 — Supplementary file1 (DOCX 1626 KB) [file 42995_2025_284_MOESM1_ESM.docx]

**
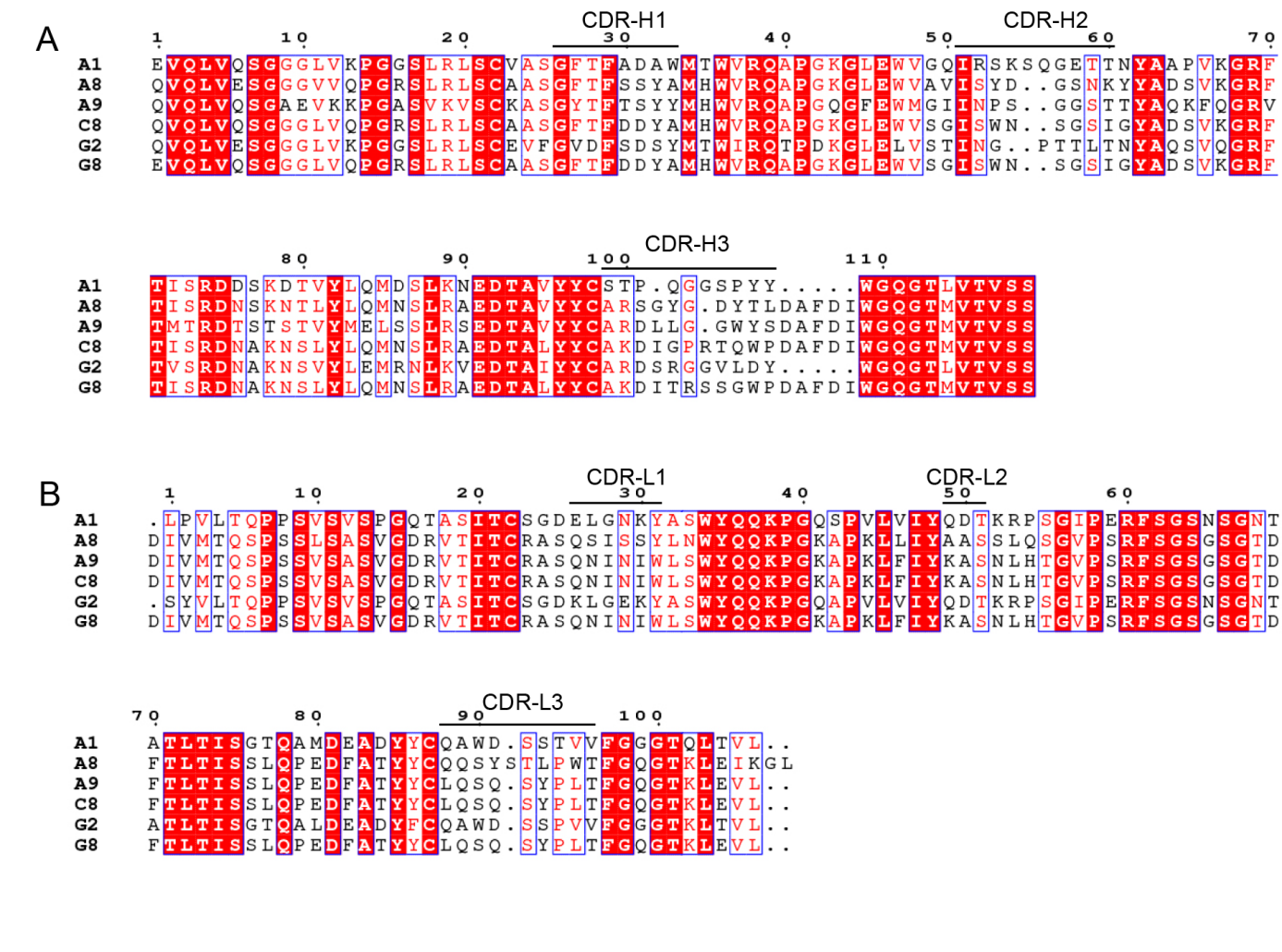
**

**Supplementary Fig. 1** Sequence analysis of anti-OX40 scFv antibodies. **A** Sequence analysis of anti-OX40 scFv antibodies heavy chains. **B** Sequence analysis of anti-OX40 scFv antibodies light chains. Figures created by ENDscript/ESPript.


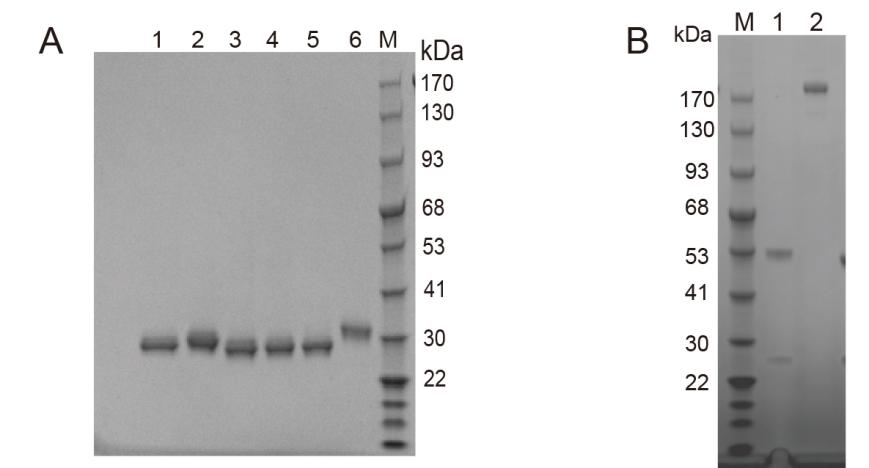


**Supplementary Fig. 2** The results of antibody SDS-PAGE. **A** Results of scFv protein isolation and purification. line1: A1; line2: A8; line3: A9; line4: C8; line5: G2; line6: G8; line M: protein marker. **B** SDS-PAGE analysis of the expression and purification processes. line M: protein marker; line 1: reduced SDS; line 2: non-reduced SDS.

**
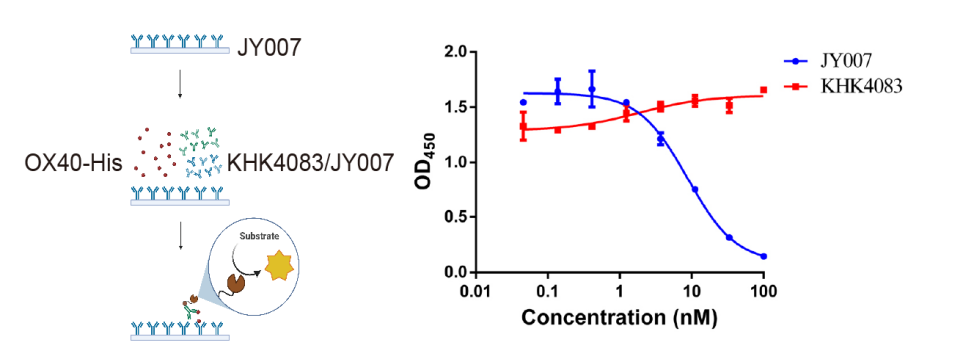
**

**Supplementary Fig. 3** Competitive ELISA between OX40, JY007, and KHK4083. Gradient dilutions of KHK4083 were incubated with precoated JY007 and 0.2 μg/mL OX40-His. Anti-His antibody was used as the secondary antibody. JY007 was used as a control. Error bars represent standard deviation. Data are presented as mean ± SD, n = 6 in triplicate. Schematic illustration created by BioRender.

**
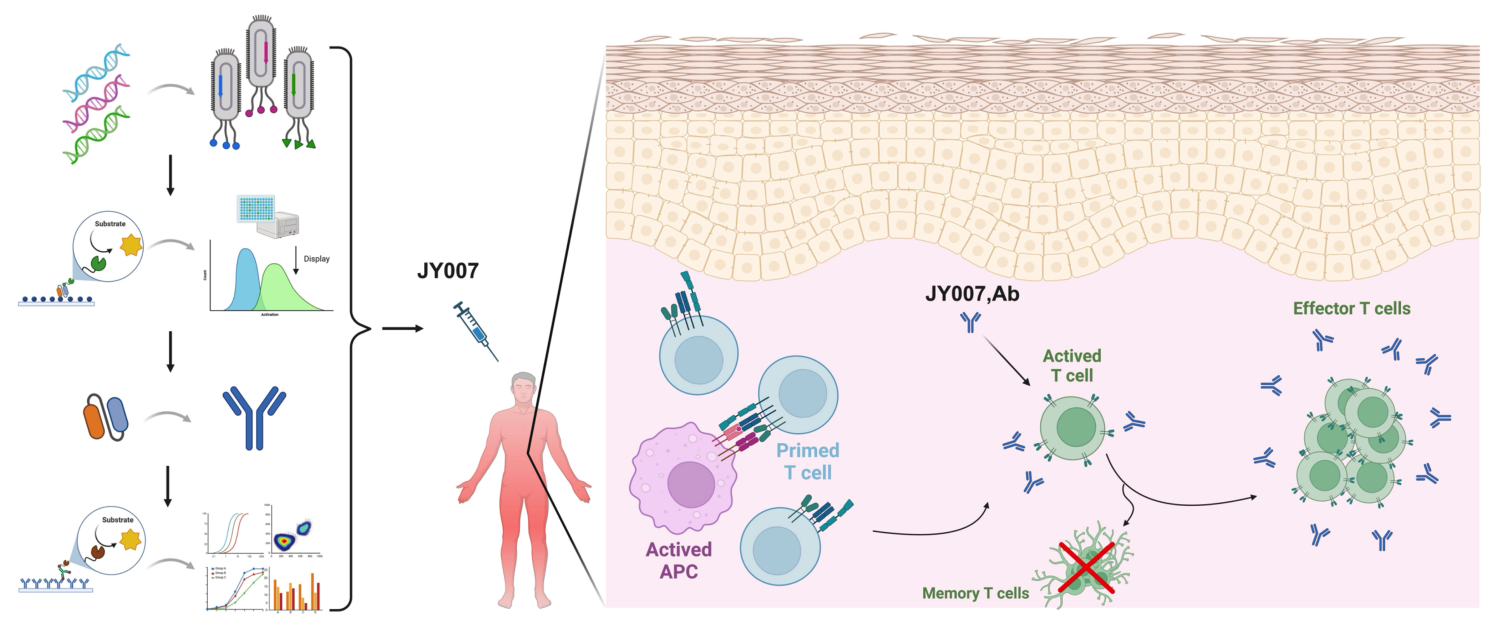
Supplementary Fig. 4** Schematic illustration of the discovery and functional studies of antibody JY007. Figure created by BioRender.

## Supplrmrntary Table 1 Amino acid sequence of OX40 wild-type and mutants

|  | Mutation Position | Sequence |
| --- | --- | --- |
| WT | NA | LHCVGDTYPSNDRCCHECRPGNGMVSRCSRSQNTVCRPCGPGFYNDVVSSKPCKPCTWCNLRSGSERKQLCTATQDTVCRCRAGTQPLDSYKPGVDCAPCPPGHFSPGDNQACKPWTNCTLAGKHTLQPASNSSDAIC |
| OX40-1 | H30A | L**A**CVGDTYPSNDRCCHECRPGNGMVSRCSRSQNTVCRPCGPGFYNDVVSSKPCKPCTWCNLRSGSERKQLCTATQDTVCRCRAGTQPLDSYKPGVDCAPCPPGHFSPGDNQACKPWTNCTLAGKHTLQPASNSSDAICEDGSHHHHHH |
| OX40-2 | D34A | LHCVG**A**TYPSNDRCCHECRPGNGMVSRCSRSQNTVCRPCGPGFYNDVVSSKPCKPCTWCNLRSGSERKQLCTATQDTVCRCRAGTQPLDSYKPGVDCAPCPPGHFSPGDNQACKPWTNCTLAGKHTLQPASNSSDAICEDGSHHHHHH |
| OX40-3 | T35A | LHCVGD**A**YPSNDRCCHECRPGNGMVSRCSRSQNTVCRPCGPGFYNDVVSSKPCKPCTWCNLRSGSERKQLCTATQDTVCRCRAGTQPLDSYKPGVDCAPCPPGHFSPGDNQACKPWTNCTLAGKHTLQPASNSSDAICEDGSHHHHHH |
| OX40-4 | Y36A | LHCVGDT**A**PSNDRCCHECRPGNGMVSRCSRSQNTVCRPCGPGFYNDVVSSKPCKPCTWCNLRSGSERKQLCTATQDTVCRCRAGTQPLDSYKPGVDCAPCPPGHFSPGDNQACKPWTNCTLAGKHTLQPASNSSDAICEDGSHHHHHH |
| OX40-5 | P37A | LHCVGDTY**A**SNDRCCHECRPGNGMVSRCSRSQNTVCRPCGPGFYNDVVSSKPCKPCTWCNLRSGSERKQLCTATQDTVCRCRAGTQPLDSYKPGVDCAPCPPGHFSPGDNQACKPWTNCTLAGKHTLQPASNSSDAICEDGSHHHHHH |
| OX40-6 | E45A | LHCVGDTYPSNDRCCH**A**CRPGNGMVSRCSRSQNTVCRPCGPGFYNDVVSSKPCKPCTWCNLRSGSERKQLCTATQDTVCRCRAGTQPLDSYKPGVDCAPCPPGHFSPGDNQACKPWTNCTLAGKHTLQPASNSSDAICEDGSHHHHHH |
| OX40-7 | R55A | LHCVGDTYPSNDRCCHECRPGNGMVS**A**CSRSQNTVCRPCGPGFYNDVVSSKPCKPCTWCNLRSGSERKQLCTATQDTVCRCRAGTQPLDSYKPGVDCAPCPPGHFSPGDNQACKPWTNCTLAGKHTLQPASNSSDAICEDGSHHHHHH |
| OX40-8 | D74A | LHCVGDTYPSNDRCCHECRPGNGMVSRCSRSQNTVCRPCGPGFYN**A**VVSSKPCKPCTWCNLRSGSERKQLCTATQDTVCRCRAGTQPLDSYKPGVDCAPCPPGHFSPGDNQACKPWTNCTLAGKHTLQPASNSSDAICEDGSHHHHHH |
| OX40-9 | V75A | LHCVGDTYPSNDRCCHECRPGNGMVSRCSRSQNTVCRPCGPGFYND**A**VSSKPCKPCTWCNLRSGSERKQLCTATQDTVCRCRAGTQPLDSYKPGVDCAPCPPGHFSPGDNQACKPWTNCTLAGKHTLQPASNSSDAICEDGSHHHHHH |
| OX40-10 | V76A | LHCVGDTYPSNDRCCHECRPGNGMVSRCSRSQNTVCRPCGPGFYNDV**A**SSKPCKPCTWCNLRSGSERKQLCTATQDTVCRCRAGTQPLDSYKPGVDCAPCPPGHFSPGDNQACKPWTNCTLAGKHTLQPASNSSDAICEDGSHHHHHH |
| OX40-11 | S78A | LHCVGDTYPSNDRCCHECRPGNGMVSRCSRSQNTVCRPCGPGFYNDVVS**A**KPCKPCTWCNLRSGSERKQLCTATQDTVCRCRAGTQPLDSYKPGVDCAPCPPGHFSPGDNQACKPWTNCTLAGKHTLQPASNSSDAICEDGSHHHHHH |
| OX40-12 | K79A | LHCVGDTYPSNDRCCHECRPGNGMVSRCSRSQNTVCRPCGPGFYNDVVSS**A**PCKPCTWCNLRSGSERKQLCTATQDTVCRCRAGTQPLDSYKPGVDCAPCPPGHFSPGDNQACKPWTNCTLAGKHTLQPASNSSDAICEDGSHHHHHH |
| OX40-13 | K82A | LHCVGDTYPSNDRCCHECRPGNGMVSRCSRSQNTVCRPCGPGFYNDVVSSKPC**A**PCTWCNLRSGSERKQLCTATQDTVCRCRAGTQPLDSYKPGVDCAPCPPGHFSPGDNQACKPWTNCTLAGKHTLQPASNSSDAICEDGSHHHHHH |
| OX40-14 | D117A | LHCVGDTYPSNDRCCHECRPGNGMVSRCSRSQNTVCRPCGPGFYNDVVSSKPCKPCTWCNLRSGSERKQLCTATQDTVCRCRAGTQPL**A**SYKPGVDCAPCPPGHFSPGDNQACKPWTNCTLAGKHTLQPASNSSDAICEDGSHHHHHH |
| OX40-15 | S118A | LHCVGDTYPSNDRCCHECRPGNGMVSRCSRSQNTVCRPCGPGFYNDVVSSKPCKPCTWCNLRSGSERKQLCTATQDTVCRCRAGTQPLD**A**YKPGVDCAPCPPGHFSPGDNQACKPWTNCTLAGKHTLQPASNSSDAICEDGSHHHHHH |
| OX40-16 | Y119A | LHCVGDTYPSNDRCCHECRPGNGMVSRCSRSQNTVCRPCGPGFYNDVVSSKPCKPCTWCNLRSGSERKQLCTATQDTVCRCRAGTQPLDS**A**KPGVDCAPCPPGHFSPGDNQACKPWTNCTLAGKHTLQPASNSSDAICEDGSHHHHHH |
| OX40-17 | K120A | LHCVGDTYPSNDRCCHECRPGNGMVSRCSRSQNTVCRPCGPGFYNDVVSSKPCKPCTWCNLRSGSERKQLCTATQDTVCRCRAGTQPLDSY**A**PGVDCAPCPPGHFSPGDNQACKPWTNCTLAGKHTLQPASNSSDAICEDGSHHHHHH |
| OX40-18 | S38A | LHCVGDTYP**A**NDRCCHECRPGNGMVSRCSRSQNTVCRPCGPGFYNDVVSSKPCKPCTWCNLRSGSERKQLCTATQDTVCRCRAGTQPLDSYKPGVDCAPCPPGHFSPGDNQACKPWTNCTLAGKHTLQPASNSSDAICEDGSHHHHHH |
| OX40-19 | D40A | LHCVGDTYPSN**A**RCCHECRPGNGMVSRCSRSQNTVCRPCGPGFYNDVVSSKPCKPCTWCNLRSGSERKQLCTATQDTVCRCRAGTQPLDSYKPGVDCAPCPPGHFSPGDNQACKPWTNCTLAGKHTLQPASNSSDAICEDGSHHHHHH |

## Supplementary Table 2 Fold decrease of KHK4083

| Mutant | Fold decrease |
| --- | --- |
| WT | 1 |
| OX40-1 | 2.99 |
| OX40-2 | 0.75 |
| OX40-3 | 0.5 |
| OX40-4 | 1.54 |
| OX40-5 | 2.94 |
| OX40-6 | NA |
| OX40-7 | NA |
| OX40-8 | 12.21 |
| OX40-9 | 0.2 |
| OX40-10 | NA |
| OX40-11 | NA |
| OX40-12 | 0.08 |
| OX40-13 | 14.81 |
| OX40-14 | 27.53 |
| OX40-15 | 6.29 |
| OX40-16 | 21.23 |
| OX40-17 | 16.3 |
| OX40-18 | NA |
| OX40-19 | 1.99 |

Note: "NA": mutants have too high affinity to be calculated.

## Supplementary Table 3 Fold decrease of JY007

| Mutant | Fold decrease |
| --- | --- |
| WT | 1 |
| OX40-1 | NA |
| OX40-2 | NA |
| OX40-3 | NA |
| OX40-4 | NA |
| OX40-5 | 10.58 |
| OX40-6 | NA |
| OX40-7 | NA |
| OX40-8 | NA |
| OX40-9 | NA |
| OX40-10 | NA |
| OX40-11 | NA |
| OX40-12 | NA |
| OX40-13 | 3.19 |
| OX40-14 | 5.27 |
| OX40-15 | 3.49 |
| OX40-16 | 1.81 |
| OX40-17 | 2.21 |
| OX40-18 | 12.24 |
| OX40-19 | 12.33 |

Note: "NA": mutants have too high affinity to be calculated.
